# Relevance between GerdQ score and the severity of reflux esophagitis in Uygur and Han Chinese

**DOI:** 10.18632/oncotarget.20146

**Published:** 2017-08-10

**Authors:** Man Wang, Jing-Zhan Zhang, Xiao-Jing Kang, Li Li, Xiao-Ling Huang, Kuerbanjiang Aihemaijiang, Aheman Ayinuer, Yue-Xian Li, Xiao-Lei He, Feng Gao

**Affiliations:** ^1^ Department of Gastroenterology, People’s Hospital of Xinjiang Uygur Autonomous Region, Urumqi, China; ^2^ Department of Dermatology, People’s Hospital of Xinjiang Uygur Autonomous Region, Urumqi, China

**Keywords:** reflux esophagitis, gastroesophageal reflux disease questionnaire, endoscopy

## Abstract

Gastroesophageal reflux disease questionnaire (GerdQ) was used to investigate the inpatients with typical reflux related symptoms in Gastroenterology. According to heartburn, regurgitation, abdominal pain, nausea, sleep disorders, whether taking over the counter (OTC) drugs 6 points to score. Using endoscopy as the gold standard for the diagnosis of reflux esophagitis (RE), and the results were compared with GerdQ score to determine the threshold value for RE, to analyze the distribution of GerdQ score for patients with RE, to assess the relationship between the GerdQ score and the severity of RE. A total of 1233 patients were enrolled in this study, including 538 patients had RE and 695 had not. There was statistical significance in the GerdQ score of RE group and non-RE group (*P* <0.05), showing that significant correlation between the score and the occurrence of RE. GerdQ score and the severity of RE were positively correlated. Further research also showed that there was a direct correlation between GerdQ score and the severity of RE in the Uygur and Han. GerdQ seems to be an useful screening tool in initial diagnosis of RE, and positively correlated with the severity of RE.

## INTRODUCTION

Reflux esophagitis (RE) is defined as reflux of the gastric and (or) duodenum contents into the esophagus causes a range of mucosal breaks, erosions or ulcers within the esophagus. RE is a prevalent, chronic and relapsing upper digestive disease, especially in recent years. With the change of people’s diet structure and lifestyle, the morbidity of RE is increasing greatly in the world [1–3], which has significantly compromised the quality of life for patients. Gastroesophageal reflux disease questionnaire (GerdQ) is designed by Dent et al [4] in 2007, it is a self-administered diagnostic questionnaire consisted by six items. It’s mainly used as a tool to improve and standardise symptom-based diagnosis and evaluation the treatment effects in patients with GERD [5]. The questionnaire is simple, convenient, non-invasive examination, low price, good patient compliance, can be completed in the clinic [6]. The diagnostic validity and reliability of GerdQ has been confirmed [7–10]. In order to understand the occurrence rates of reflux related symptoms, to explore the correlation between the endoscopic manifestations and GerdQ score, and to provide reference for clinical diagnosis of RE, we conducted the study.

## RESULTS

### General characteristics of the patients

**Figure 1:**
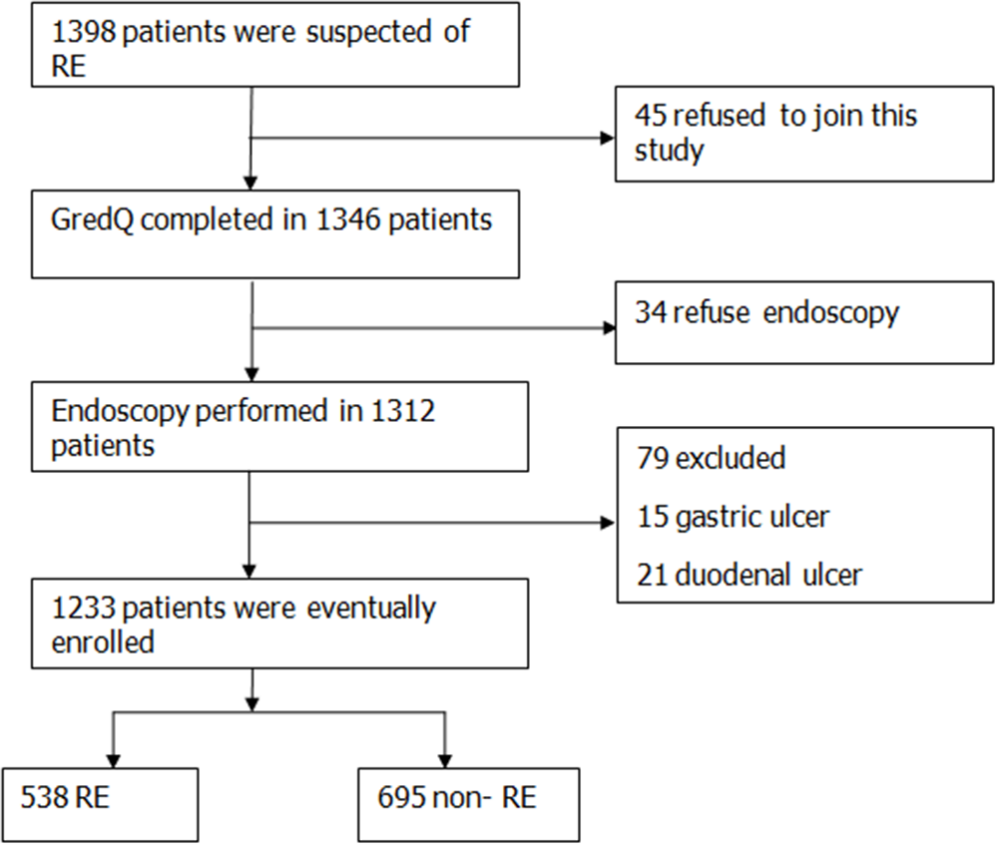
Flow chart of the patients’ enrollment

A total of 1398 patients were collected for the study, of whom 1233 were enrolled in the final analysis. A flowchart of the study, subject withdrawal at various stages and the final diagnoses are shown in Figure 1. Among the 1233 patients, 532 were males and 701 were females. Their mean age was 53.72±11.99 years (range 18-81 years). Their mean body mass index (BMI) was 25.34 ± 3.73 kg/m^2^. There were 482 were Uygur and 751 were Han patients, among whom 538 were RE and 695 were non-RE. In Uygur, 234 were RE patients and 248 were non-RE. In the Han, 304 cases were RE, and 447 were non-RE. The basic characteristics of these patients in RE group and non- RE group were shown in Table 1. The incidence of male patients in the RE group was significantly higher than male patients in the non-RE group (*χ*^*2*^=23.567, *P*<0.01). There were no difference between the RE and the non-RE groups in age (*t*=-0.884, *P*>0.05), BMI (*t*=1.190, *P*>0.05) and educational level (*χ*^*2*^=0.307, *P*>0.05), but 83.09% RE patients had education level beyond secondary school. The Uygur and Han in the RE group and non-RE group account for the proportion had significant difference (*χ*^*2*^=7.771, *P*<0.01), the detection rate of RE in the Uygur was higher than that in the Han.

**Table 1:** Characteristic of RE group and non-RE group

Items	RE	Non-RE	Test statistic	*P* value
Total number (n)	538	695		
Gender, Male [n (%)]	274 (50.90)	258 (37.08)	*χ*^*2*^=23.567	0
Age (mean±SD, yr)	53.46±12.28	55.13±10.34	*t*=-0.884	0.378
BMI (mean±SD)	25.45±3.74	24.72±3.66	*t*=1.190	0.235
Ethnic [n(%)]			*χ*^*2*^=7.771	0.005
Uygur	234 (48.55)	248 (51.45)		
Han	304 (40.48)	447 (59.52)		
Education level [n(%)]			*χ*^*2*^=0.307	0.858
Primary school	91 (16.91)	120 (17.26)		
Secondary school	267 (49.63)	334 (48.06)		
College	180 (33.46)	241 (34.68)		

### The GerdQ cut-offs

It is noted in patients with a GerdQ sum score between 0 and 14, the RE show direct correlation with the increasing of GerdQ cut-off scores. In those with a sum score of 8-10, 61.01% had RE, and in those with a sum score of 3-7, 15.94% had RE. However, none with a score of 0-2 had RE.

### RE classification and GerdQ scores

Of the 538 patients with RE, nearly 412 cases (76.58%) were demonstrated as grade A lesion, 76 cases (14.12%) were categorized as grade B, 32 cases (5.95%) were grade C, and only 18 cases (3.35%) were grade D lesion. The distribution of GerdQ scores in all patients is shown in Table 2. The mean GerdQ score of patients with RE and non-RE were 10.08±2.89 and 9.11±3.52, respectively, and had significant difference between the two groups (*t*=2.33, *P*<0.05). All of the RE patients were graded according to LA classification, showed that the mean GerdQ scores of patients with LA-A, LA-B, LA-C and LA-D was 10.48±2.91, 11.75±2.77, 11.80±2.98 and 13.35±2.95, respectively. Thus it can be seen, the total GerdQ score increased with increasing severity of esophageal mucosal defect. Spearman correlation analysis showed that the GerdQ score was positively correlated with the severity of RE (*r*=0.244, *P*<0.01). The mean GerdQ score of patients with Uygur and Han was 10.23±3.17 and 10.03±2.72, respectively, Spearman rank correlation analysis showed that there was a direct correlation between the GerdQ score and the severity of RE in the Uygur and Han (*r*=0.233, *P*<0.05; *r*=0.201, *P*<0.05).

**Table 2:** Relationship of RE grade and symptomatic scores

Grading of RE	Cases (n)	Range	GerdQ score
The non-RE	695	2∼18	9.11±3.52
The RE	538	3∼18	10.08±2.89
LA grade A	412	3∼18	10.48±2.91
LA grade B	76	6∼18	11.75±2.77
LA grade C	32	6∼18	11.80±2.98
LA grade D	18	7∼17	13.35±2.95

### Diagnostic value of the GerdQ

**Figure 2:**
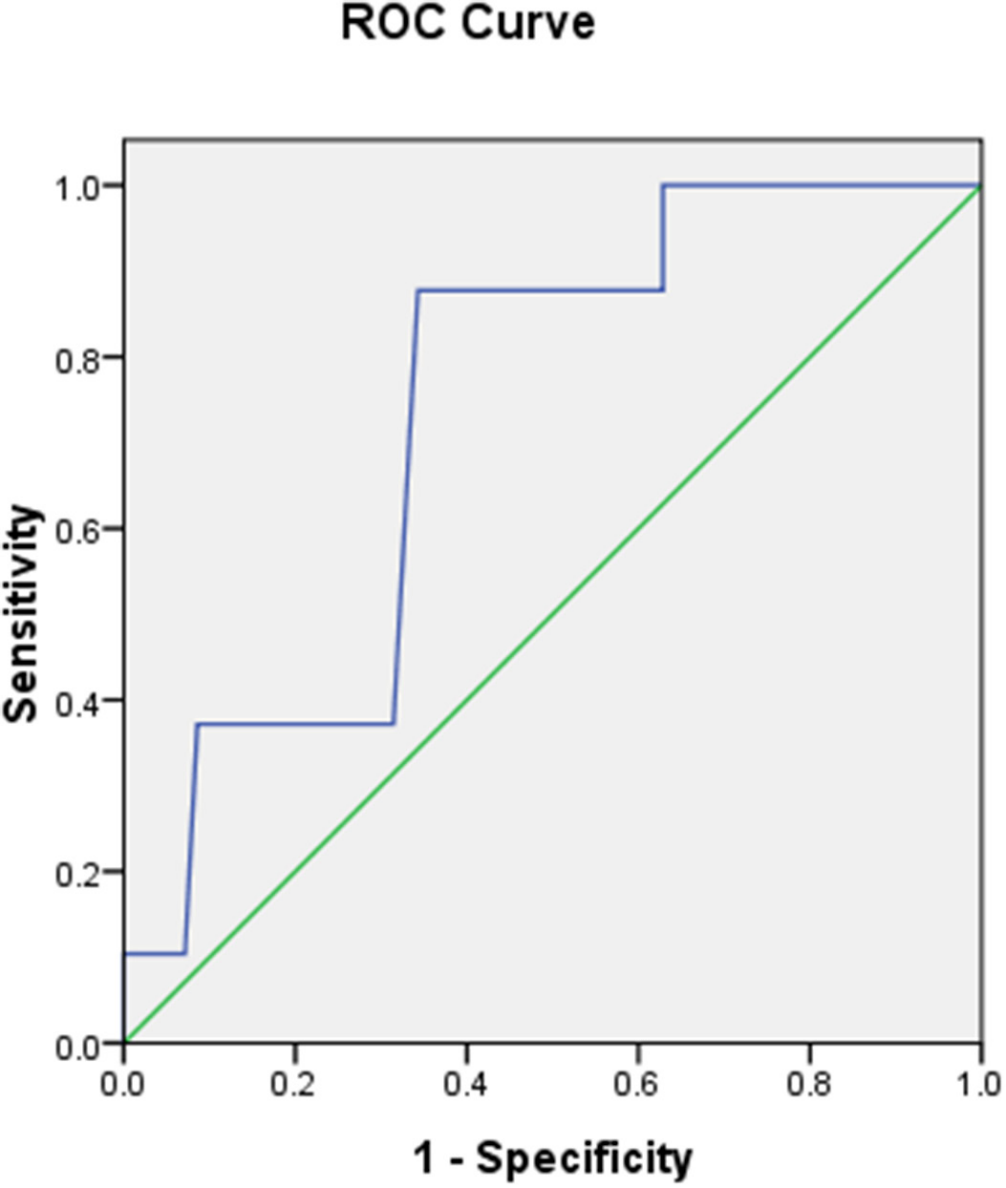
ROC curve of the GerdQ Specificity and sensitivity by cut-off score.

With the increasing of critical value of GerdQ, the sensitivity decreased gradually, and the specificity increased. Suggesting that Gerd Q is a good diagnostic tool. When the cut-off score was 9, the Youden index reached a maximum of 0.53, in which the area under the ROC was 0.736, the ROC analysis gave the optimal balance between sensitivity (87.7%) and specificity (65.7%) for RE, it showed that the questionnaire had high credibility (Figure 2).

## DISCUSSION

RE is a chronic, recurrent disease commonly recognized in developed countries and developing countries. The disease manifests itself with varying severity, from mild to incapacitating, with significant impairment of quality of life [12]. The cardinal symptoms are considered to be heartburn and regurgitation. Furthermore, the burden of the disease is large and affects the quality of life for patients, resulting in both direct health care related costs as well as indirect costs due to loss [13]. RE is a symptom based disease, and more and more people pay attention to the symptom based questionnaire. GerdQ scale provides us a simple, reliable and effective diagnostic method, and it has high clinical application value.

Among the 538 patients with RE, including 274 males, the proportion of males was higher than that of males with non-RE (including 258 males), showing that male patients are more likely to develop into RE, which is consistent with Vakil N et al [14] and Hu et al [15]. This suggested that we should strengthen management of male patients to reduce the incidence of RE. Compared with the non-RE group, 83.09% patients with the level of education were Secondary school or above in the RE group. The result was consistent with the research of Bai et al [7], which indicates that people with higher educational level are more concerned about their own health. Suggesting that we should strengthen the popularization of RE related medical knowledge, seminars, Conduct lectures, free medical consultation clinic and other propagandas, to improve the patient's awareness of RE.

On the other hand, the incidence of RE in Uygur patients was significantly higher than that in Han, suggesting that the characteristics of RE in different ethnic groups were different. Munila et al [16] conducted a survey of the prevalence of GERD in the Han and Uygur physical examination population and found that the incidence rate of GERD in Uygur was higher than that in Han. This difference may leaded by different national culture, religious belief, lifestyle and eating habits. The Uygur often eat more carnivorous, love oil Nang, pilaf, tea and other greasy food, the Uygur men often drink (liquor based), smoking is also more common, these are likely to affect the incidence. Secondly, because the GerdQ symptom questionnaire itself factors, different patients have different understanding and expression of heartburn, acid reflux symptoms. As well as the impact of local language and education, which may have a certain impact on the results of the GerdQ score.

The proportion of patients with RE group rise up with the increasing of GerdQ score from 0 to 14, which Bai's [7] research had the same results. So patients with more severe reflux symptoms are more likely to have RE. Further study showed that the risk of RE will be relatively small when the GerdQ total score <8, and none of the patients with a GerdQ score of 0–2 had RE. This finding is in agreement with previous study [5]. It shows that the lower the GerdQ score, the smaller of the risk of suffering from RE. A higher score signifies a greater possibility of RE.

This study shows that GerdQ average score is higher in the RE group than that in the non-RE group, the score of esophageal erosion was significantly higher than that of the normal controls, GerdQ score has a good value to distinguish the RE group and the non-RE group. The higher the score, the greater possibility of suffering from RE.

For the RE patients, the majority of them (90.71%) were suffering from grade A or grade B, and this result is consistent with the results of Ma et al [17], which found 96.9% of Chinese patients with RE had LA grade A or grade B lesion. This suggests RE patients tend to be less severe in Chinese patients than it is in Western population. The study also showed that the GerdQ score increased with increasing grade of RE. Spearman rank correlation analysis showed that the GerdQ score was positively correlated with the severity of RE (P<0.01). Further study showed that there was a direct correlation between the GerdQ score and the severity of RE in the Uygur and Han. That is, the higher the score, the more severe esophageal mucosal erosion. The result is same to the Uygur and Han. It indicated that the symptom score of RE has clinical diagnostic value, we can rely on the GerdQ score to distinguish the RE grading. It is consistent with the results of Zhai et al [18]. But Li et al [19] and Pace et al [20] found that there was no correlation between GerdQ score and severity of RE. The reasons for the difference may be related to the ethnic and geographical differences of the subjects in the study. This study still has a guiding significance for the diagnosis of RE in Chinese population.

We found that the optimal GerdQ cutoff score for RE in our study was 9, corresponding to a sensitivity of 87.7% and a specificity of 65.7% for the diagnosis of RE, which is the same as the study of Jonasson *et al* [8], who reported a sensitivity of 66% and a specificity of 64% if the cut-off value of GerdQ score is increased to 9. However, in a previous studied by Jones *et al* [5] indicated that the sensitivity and specificity were 65% and 71%, respectively, the diagnosis of GERD with a GerdQ cut-off value of ≥8, and other studies have shown a sensitivity for GerdQ of 57–78% and specificity of 46–50% for the diagnosis of GERD when the GerdQ score was ≥8 [21, 22, 8]. The difference in the sensitivity and specificity of GerdQ for the diagnosis of RE may be attributed to the following reasons: In this study, all the patients enrolled were Uygur and Han in Xinjiang region, the difference of the way of life, economic status, geographical environment and other factors.

The present study has several strengths: (1) There were many studies about the application of GerdQ score in the diagnosis of gastroesophageal reflux disease, but no independent study about the correlation between GerdQ score and the severity of RE, our study will be useful in the diagnosis of RE, for which current data is largely lacking; (2) This study analyzed the correlation between GerdQ score and the severity of RE for Uygur and Han come to the same conclusion, which made the conclusion more convincing. Therefore, our findings are of great significance in guiding clinical practice.

## MATERIALS AND METHODS

### Study population

Data from consecutive Uygur and Han patients who were suspected of RE in the medical centre of the People’s Hospital of Xinjiang Uygur Autonomous Region were prospectively collected from August 1, 2014 to December 31, 2015. Inclusion criteria: (1) Patients with heartburn and (or) regurgitation as the main symptoms manifested over the last 4 weeks; (2) Patients with symptoms suggestive of RE, who underwent first diagnostic; (3) Adult (age≥18 years) male or female; (4) Educational level above elementary school, able to fill out the questionnaire independently; (5) Written informed consent. Exclusion criteria: (1) Patients with alarm symptoms such as weight loss, dysphagia, gastrointestinal bleeding, anemia, hematemesis, melena, etc; (2) Take any proton pump inhibitors, histamine 2 receptor antagonists, or gastrointestinal motility drugs for more than 2 weeks before inclusion; (3) Underwent upper gastrointestinal bleeding, a history of gastric surgery, esophageal stenosis, peptic ulcer, gastric, esophageal varices, or malignant tumors in 1 year; (4) Gastroscopy contraindications or refusing endoscopy ; (5) Patients with severe cardiovascular, pulmonary, liver and kidney diseases, or other severe mental diseases; (6) Pregnancy and lactating women; (7) History of taking non steroidal anti-inflammatory drugs; (8) History of alcohol or drug abuse; and (9) patients were diagnosed with RE and during treatment.

### Study design

Research steps: All of the Eligible patients provided written informed consent followed by completion of the GerdQ, blinded to the investigator, and a GerdQ sum score was calculated. Endoscopy was then performed. GerdQ was completed blinded to the endoscopic doctor. The patient who had esophagocardiac mucosal erosions were detected by endoscopy would be classified according to the Los Angeles classification system. The study protocol was approved by the Ethics Review Committee of Xinjiang Uygur Autonomous Region of China.

Questionnaire: The questionnaires, which comprised the General data questionnaire and the Chinese version of GerdQ, demographic information (gender, age, ethnic, height, weight, educational level and so on). The GerdQ is a symptom scale (Table 3), which including heartburn, regurgitation, epigastric pain, nausea, sleep disturbance, and use of over the counter (OTC) drugs. Patients were asked to review the frequencies of various symptoms during last week, and which were described by a Likert scale from 0 to 3 for positive symptom problems and from 3 to 0 for negative symptom problems, with a total GerdQ score range from 0 to18.

**Table 3:** The GerdQ questionnaire

Questions	Frequency score (points) for symptoms			
	0 day	1 day	2∼3days	4∼7days
How often did you have a burning feeling behind your breastbone (heartburn)?	0	1	2	3
How often did you have stomach contents (liquid or food) moving upwards to your throat or mouth (regurgitation)?	0	1	2	3
How often did you have pain in the center of the upper stomach?	3	2	1	0
How often did you have nausea?	3	2	1	0
How often did you have difficulty getting a good night’s sleep because of your heartburn and/or regurgitation?	0	1	2	3
How often did you take additional medication for your heartburn and/or regurgitation, other than what the physician told you to take (such as Tums, Rolaids and Maalox)?	0	1	2	3

Endoscopy: All patients were required to fast for at least 12 h prior to the conventional endoscopy (Olympus-260, Tokyo, Japan). The severity of RE was graded according to the Los Angeles classification system (LA grading) [11]. Grade A: one or more mucosal break less than 5mm long, which doesn't extend between the tops of two mucosal folds. Grade B:one or more mucosal break more than 5mm long, which doesn't extend between the tops of two mucosal folds. Grade C: one or more mucosal break that is continuous between the tops of two or more mucosal folds but which involves less than 75% of the oesophageal circumference. Grade D: one or more mucosal break which involves more than 75% of the oesophageal circumference.

The diagnostic standard of RE: Take endoscopy as the gold standard for the diagnosis of RE. All participants were divided into patients with RE and those with non-RE according to whether they had or had not esophagocardiac mucosal break or erosions found by endoscopy.

### Statistical analysis

Statistical analyses were performed using SPSS software (version 17.0). All data are expressed as mean ± standard deviation (SD), percentages and ranges. The measurement data and numeration data were assessed by t-test and chi-square test respectively. and Spearman rank correlation analysis was used for ranked data. ROC curve was used to determine the optimal cut-off for the diagnosis of RE. In all analyses, *P* < 0.05 was considered statistically significant.

## CONCLUSIONS

The GerdQ score has good value to distinguish RE patients from non-RE patients. GerdQ score was positively correlated with the severity of RE. The higher of the score, the more severe of the esophageal mucosal erosion. Further analysis for the Uygur and Han we both come to the same conclusion. Although RE cannot be accurately diagnosed by the GerdQ score alone in patients suspected of RE, it can be regarded as a useful tool for screening RE in the general population. A definitive diagnosis of RE still depends on endoscopy.
